# Origin, Diversity, and Multiple Roles of Enzymes with Metallo-β-Lactamase Fold from Different Organisms

**DOI:** 10.3390/cells12131752

**Published:** 2023-06-30

**Authors:** Seydina M. Diene, Pierre Pontarotti, Saïd Azza, Nicholas Armstrong, Lucile Pinault, Eric Chabrière, Philippe Colson, Jean-Marc Rolain, Didier Raoult

**Affiliations:** 1 MEPHI, IRD, AP-HM, IHU-Méditerranée Infection, Aix Marseille University, 13005 Marseille, France; 2 IHU-Méditerranée Infection, 13005 Marseille, France; said.azza@univ-amu.fr (S.A.);; 3 CNRS SNC5039, 13005 Marseille, France; 4 Assistance Publique-Hôpitaux de Marseille (AP-HM), IHU-Méditerranée Infection, 13005 Marseille, France

**Keywords:** metallo-β-lactamase (MβL) fold proteins, multifunctional enzymes, antibiotic-hydrolysing activity, nuclease, ribonuclease, lactonase, domains of life

## Abstract

β-lactamase enzymes have generated significant interest due to their ability to confer resistance to the most commonly used family of antibiotics in human medicine. Among these enzymes, the class B β-lactamases are members of a superfamily of metallo-β-lactamase (MβL) fold proteins which are characterised by conserved motifs (i.e., HxHxDH) and are not only limited to bacteria. Indeed, as the result of several barriers, including low sequence similarity, default protein annotation, or untested enzymatic activity, MβL fold proteins have long been unexplored in other organisms. However, thanks to search approaches which are more sensitive compared to classical Blast analysis, such as the use of common ancestors to identify distant homologous sequences, we are now able to highlight their presence in different organisms including Bacteria, Archaea, Nanoarchaeota, Asgard, Humans, Giant viruses, and Candidate Phyla Radiation (CPR). These MβL fold proteins are multifunctional enzymes with diverse enzymatic or non-enzymatic activities of which, at least thirteen activities have been reported such as β-lactamase, ribonuclease, nuclease, glyoxalase, lactonase, phytase, ascorbic acid degradation, anti-cancer drug degradation, or membrane transport. In this review, we (i) discuss the existence of MβL fold enzymes in the different domains of life, (ii) present more suitable approaches to better investigating their homologous sequences in unsuspected sources, and (iii) report described MβL fold enzymes with demonstrated enzymatic or non-enzymatic activities.

## 1. Introduction

Initially discovered in bacteria due to their effectiveness against antibiotics with therapeutic interest in humans, β-lactamases are a group of enzymes capable of degrading several β-lactam antibiotics [1,2]. They are a typical example of the artificial naming of enzymes that have, in reality, multiple potential functions, and this nomenclature has prevented the exploration of their activities and their presence in other organisms or microorganisms. Furthermore, the nomenclature used to describe proteins of the same family may vary depending on the method adopted by researchers, thus enzymes belonging to the same family may be labelled as ribonucleases (RNases), nucleases (DNases), hydrolases, or β-lactamases, depending on the automatic protein annotation, which relies on the initial sequence hits obtained through Blast analysis. The study of the ancestry of β-lactamase motifs has shown that they exhibit some of the oldest enzymatic motifs in the world [3]. Once the antiquity of this type of enzyme has been recognised, the search for sequences which are unrelated to this group of β-lactamases becomes more difficult, due to their early divergence from the ancestral structure. As such, the use of more sensitive approaches (compared with classical Blast analysis) to search for distant homologous sequence, such as the reconstruction of a common ancestor sequence, as previously reported for class A β-lactamases [4,5] and using it as a query makes it possible to identify sequences that are too far away from contemporary sequences to be identified in a functional way.

In this review, we aim to summarise the results of this approach, which we initiated a few years ago to analyse the functions, origin, and distribution of this group of enzymes, especially metallo-β-lactamases (MβL), that have multiple more or less specialised functions depending on the ecosystem within which they are established [6]. Here, we discuss the presence of these enzymes in bacteria, archaea, candidate phyla radiation (CPR), giant viruses, and eukaryotes, including human cells and the means of expressing them and analysing their roles, which are much broader than cutting out the β-lactams used in human medicine.

## 2. Diversity of the Superfamily of Metallo-β-Lactamase (MβL) Fold Enzymes

Within the classification of bacterial β-lactamases, which consist of four classes: A, B, C, and D [7,8], MβL enzymes alone occupy class B which is subdivided into three subclasses (subclass B1/B2/B3) as the result of divergent evolution events. They differ from the other classes by their active site which requires bivalent metal ions (such as Zn^2+^, Fe^2+,^ Mg^2+^, or Ca^2+^) for their activity [7,9,10,11]. This catalytic site, characterised by a highly conserved motif (HxHxDH) and residues (H196 and H263), appears to be ancestrally shared by the superfamily of MβL fold proteins (with 34,000 proteins identified to date) with diverse biological functions including β-lactamases, nucleases, ribonucleases, lactonases, glyoxalases, hydrolases, phosphodiesterases, Aryl sulfatases, Alkylsulfatases, CMP-NeuAc hydroxylases, flavoproteins, and others which are distributed across the different domains of life including bacteria, archaea, and eukaryotes [12,13,14]. Moreover, as reported, the multifunctionality of these MβL fold enzymes and their affinities to different substrates are supported by the protein variable region of the enzymes (rather than the conserved motif (i.e., HxHxDH) and cofactor dependencies, which are responsible for the modulation of enzymatic activity, specificity, and oligomerisation proteins [10,15]. However, the protein similarity between enzymes of this MβL fold superfamily can be less than 20% [13,16] making it very unlikely that homologous sequences of contemporary bacterial MβLs in the other microorganisms such as Archaea, Giant viruses, Asgard, Nanoarchaeota, or Candidate Phyla Radiation (CPR) will be found. As reported in the “Darwinian Grandparenting” theory, we are genetically closer to our grandparents than to our cousins [17]; thus, the use of inferred common ancestor sequences as queries to identify homologous bacterial MβLs from not-yet investigated microorganisms represents one of the more pertinent approaches to identifying these enzymes in any domain of life. More sensitive approaches, including reconstruction of the common ancestor sequence [5,18], searching for Hidden Markov Models (HMM) profiles [18,19], Sequence Similarity Network analysis (SSN) [13,20], and 3D structure similarity analysis, have been used to look for homologous MβL enzymes in remote sources. As expected, these approaches appear significantly more flexible than classic Blast analysis in terms of identifying homologous MβL enzymes in unsuspected organisms and/or microorganisms.

## 3. MβL Fold Enzymes in Bacteria: Class B β-Lactamases

### 3.1. Distribution and Diversity of MβL Fold Enzymes in Bacteria

**Figure 1:**
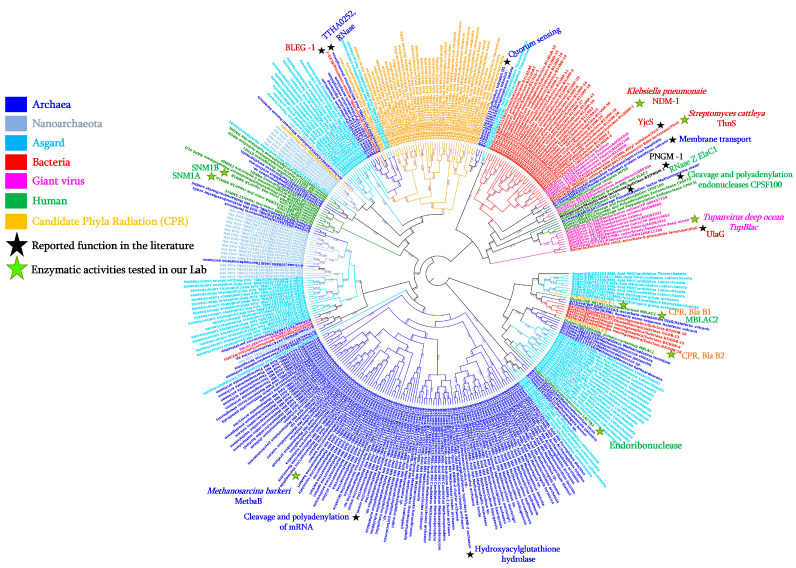
Phylogenetic tree of MβL fold enzymes from the different domains of life, including Bacteria, Archaea, Asgard, Nanoarchaeota, Giant viruses, Candidate Phyla Radiation, and Humans.

In bacteria, these MβL enzymes include more than 325 variants grouped into 63 MβL types and divided into three sub-groups: subgroup B1 (e.g., NDM-1, VIM-2, and IMP-1); sub-group B2 (e.g., CphA1, CphA7, and ImiS); sub-group B3 (e.g., GOB-13, LRA-1, and CAR-1) (Figure 1), based on their differences in amino acid sequences and their catalytic sites which interact with either one or two Zn^2+^ ions [21,22,23]. The metallo-β-lactamase enzyme was identified for the first time in 1966, in a non-pathogenic strain of *Bacillus cereus* with a cephalosporinase activity which was inhibited when pre-incubated with ethylene-diamine-tetra-acetic acid (EDTA) [24]. Nowadays, MβL enzymes can be identified in more than 50 different bacterial species including gram-negative bacteria such as Enterobacteriaceae spp. and non-fermentative bacteria (such as, *Acinetobacter* spp. and *Pseudomonas* spp.), *Elizabethkingia* spp., *Stenotrophomonas* spp., *Neisseria* spp., *Aeromonas* spp., *Shewanella* spp., *Myroides* spp., *Pedobacter* spp., *Empedobacter* spp., *Bacteroides* spp., *Vibrio* spp., *Bradyrhizobium* spp., *Caulobacter* spp., *Eristalis* spp., *Sphingomonas* spp., *Massilia* spp., *Burkholderia* spp., *Pectobacterium* spp., and *Gemmatimona* spp.) [21,22,23]. Interestingly, as shown in Figure 1, while all the bacterial B1 and B2 MβL enzymes group together separately to the other domains of life, the GOB type enzymes (sub-group B3), are strongly related with enzymes from archaea and archaea-related microorganisms (i.e., Asgard), suggestive of their origin from this domain of life, and a horizontal transfer has occurred from archaea to a single bacterial group, namely, the *Flavobacteriacaea* family, especially in *Elizabethkingia* species. All the bacterial MβLs presented in this phylogenetic tree have been reported with proof of hydrolase activity on β-lactams, especially on carbapenems, and are all described in gram-negative bacteria except one MβL type i.e., BcII MβL enzyme (with seven variants), reported in a single bacterial gram-positive species, *Bacillus cereus* [21,25]. However, based on the MβL signature, these proteins have also been identified in other Gram-positive bacteria such as *Streptococcus pneumoniae*, in which MβL fold proteins are recognised as choline-binding proteins, DNA uptake-related proteins (nucleases) and L-ascorbate 6-phosphate lactonase [26,27,28]. Interestingly, it has been reported that the enzymatic characterisation of the L-ascorbate 6-phosphate lactonase enzyme from *S. pneumoniae* ATCC 49136 shows a β-lactamase activity since the purified enzyme is able to hydrolyse both nitrocefin and ampicillin-based antibiotics [27].

### 3.2. Reported Activities of Bacterial MβL Enzymes Other Than β-Lactams Hydrolysis

Besides their hydrolytic activities on β-lactam antibiotics, some bacterial MβL enzymes have been reported with other enzymatic activities as a result of the striking similarity between their protein structures and other enzymes including ribonuclease, nuclease, and lactonase enzymes. Indeed, as reported recently, the classical bacterial MβL IMP-1 enzyme, which hydrolyses all β-lactams including carbapenems, exhibits significant protein structure similarity with tRNase Z, a tRNA 3′ processing endoribonuclease of the MβL superfamily from *Thermotoga maritima*. Its enzymatic characterisation demonstrates a significant RNA-hydrolysing activity on both cellular RNA and synthetic small unstructured RNAs [29]. Interestingly, while this study was being published, our research was beginning to reveal the ribonuclease and nuclease ability of the IMP-1 homologous enzyme i.e., class B NDM-1, described in almost all gram-negative bacteria, which significantly hydrolyses in vitro bacterial RNA and single-strand DNA substrates (Supplementary Figure S1). Moreover, while both bacterial MβL enzymes mentioned above can interact with RNA and/or DNA, others such as the ThnS enzyme can exhibit additional activities, such as the hydrolysis of ascorbic acid, as a result of its similarity with UlaG enzymes [30]. Indeed, as we reported recently, while the *thnS* gene, part of the thienamycin (now chemically modified into imipenem in human medicine) biosynthesis gene cluster from *Streptomyces cattleya*, is annotated as putative β-lactamase with no reported proof of this activity. We demonstrated its specific hydrolase activity and UlaG high affinity with imipenem in comparison with the other β-lactams (e.g., penicillin G and cefotaxime). As a result of the phylogenetic tree and conserved motif analyses, the ThnS enzyme appears to be a member of the superfamily of MβL fold enzymes, showing additional activities of ribonuclease, nuclease, and hydrolysis of ascorbic acid [30].

Recently, an MβL fold enzyme (BLEG-1) has been reported in the *Bacillus lehensis* G1 strain, exhibiting significant sequence similarity and activity with the B3 subclass of bacterial MβLs, despite its evolutionary divergence from them [31,32] (Table 1). Upon analysing the phylogenetic tree and comparing the protein structures, it was discovered that the enzyme possessed an active site that was remarkably similar to those found in both the L1 B3 MβL from *Stenotrophomonas maltophilia* and the glyoxalase II enzymes (YcbL and GloB) from *Salmonella enterica.* Interestingly, the enzymatic characterisation of the purified BLEG-1 protein demonstrates its dual β-lactams hydrolysis (e.g., ampicillin hydrolysis) and glyoxalase activities [31]. The authors identify an insertion of two amino acids into the active-site loop at the N-terminal region of the BLEG-1 protein and suggested an evolution of the BLEG-1 enzyme from glyoxalase II to the adopted MβL fold activity through this insertion of amino acids [31].

Recently, another atypical enzymatic activity of two MβL fold proteins has also been described from a functional metagenomic study of forest soil [34]. In this study, while the authors performed a function-based screening of libraries generated from the whole metagenomic sequence data of forest soil to identify positive phytase activity in *E. coli* clones, two clones were positive for this phytase activity. Surprisingly, while phytic acid degradation activity has been restricted to only four protein superfamilies, including histidine phosphatases, tyrosine phosphatases, purple acid phosphatases, and β-propeller phosphatases [47,48], the two obtained proteins (MβLp01 and MβLp02) from this metagenome were annotated and identified as genes encoding for metallo-β-lactamase proteins. Sequence analysis confirmed their membership of the MβL fold superfamily of proteins due to their close protein structure homology with the MβL ZipD from *E. coli*, a zinc phosphodiesterase with a tRNA-processing endonuclease activity [49]. Based on this discovery, the two proteins were subcloned, expressed, and enzymatically tested. As expected, the enzymatic characterisation revealed for both purified proteins an activity on the majority of tested phosphorylated substrates including phytate. Moreover, both purified enzymes were able to confer to recombinant *E. coli* strains less sensitivity to β-lactam antibiotics, suggestive of a β-lactamase activity, and qualified by the authors as promiscuous activity [34]. This promiscuous β-lactamase activity was also reported from the discovered and identified subclass B3 MβL protein, PNGM-1 from a conducted functional metagenomic analyses of deep-sea sediments predating the era of antibiotics [35,50]. Indeed, the phylogenetic and protein structure analyses of the PNGM-1 protein revealed its membership of the MβL fold superfamily and its structural similarity with the tRNA Z enzyme, and the activities test confirmed a dual enzymatic β-lactamase and ribonuclease activity of this PNGM-1 protein [35].

Another example of bacterial MβL fold enzymes with activities other than β-lactam hydrolase is that of lactonase enzymes. Recognised as members of the metallo-β-lactamase superfamily as a result of their conserved HxHxDH MβL motif [51,52], these enzymes are involved in the bacterial quorum sensing mechanism, especially by disrupting bacterial signalling via the enzymatic degradation of acyl homoserine lactone (AHL) molecules [14,52]. The quorum sensing mechanism is described as a communication system used by bacteria to manage large panels of biological processes often related to pathogenicity, such as the production of proteases, or antimicrobial compounds, such as violacein [53]. These enzymes have been reported in various and atypical bacterial species such as thermoacidophilic bacteria including *Alicyclobacter acidoterrestris*, *Geobacillus caldoxylosilyticus*, *Chromobacterium* spp., *Bacillus thuringiensis*, *Escherichia coli*, *Ruegeria mobilis*, *Microbacterium testaceum*, *Muricauda olearia*, *Arthrobacter* sp. and *Chryseobacterium* spp. [28,51,52,53,54,55,56,57,58,59].

## 4. MβL Fold Enzymes in Archaea

### 4.1. Reconstruction of Common Ancestral Sequences and Blast Analyses

In our previous research, to investigate MβL sequences from unsuspected microorganisms such as archaea, which are naturally resistant to all antibiotics including β-lactams because they lack peptidoglycans, we performed the reconstruction of ancestral bacterial β-lactamases, especially class B metallo-β-lactamases based on the maximum likelihood phylogenetic tree analysis using 174 class B MβL variants retrieved from the Arg-annot database [60]. Using an inferred MβL fold ancestor sequence as the query term in a BlastP analysis performed against the archaea database, we were able to identify a huge number of archaeal MβL fold sequences, while BlastP analysis, using contemporary bacterial sequences did not detect these archaeal sequences [6]. All detected MβL fold sequences exhibited the MβL signature (i.e., the conserved “HxHxDH” motif) and are widely distributed in different archaeal groups including *Methanomicrobia*, *Thermococci*, *Archaeoglobi*, *Methanococci*, *Thermoplasmata*, *Thermoprotei*, *Methanobacteria*, *Thaumarchaeota*, and *Asgardarchaeota* (Figure 1).

### 4.2. The Presence of MβL Fold Enzymes in Subgroups of Archaea: Nanoarchaeota and Asgard Groups

**Figure 2:**
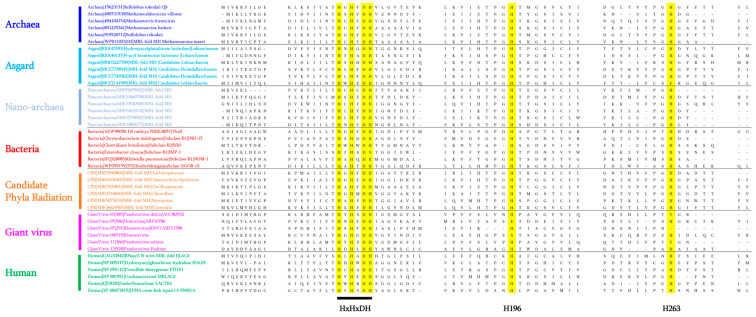
Protein sequence alignment highlighting conserved motif (“HxHxDH”) and residues (H196 and H263) from the catalytic site of MβL fold enzymes.

As proof of concept, we once again used a MβL fold ancestor sequence (120 aa in size) from a constructed Maximum Likelihood phylogenetic tree with sequences from bacteria, archaea, humans, and CPRs to confirm the existence of these enzymes in archaea-related groups such as Asgard and Nanoarchaeota. As expected, by conducting a BlastP analysis using the inferred MβL fold ancestor sequence as the query term, we were able to detect the presence of these MβL fold enzymes in these groups of microorganisms (Supplementary Tables S1 and S2). In the Asgard group, MβL fold sequences exhibited protein similarity of between 25% and 46.25%, and alignment of between 48% and 100% with the ancestral MβL fold protein sequence. These were distributed across different Asgard sub-groups such as *Helarchaeota*, *Lokiarchaeota*, *Heimdallarchaeota*, *Thorarchaeota*, *Lokiarchaeum*, and *Odinarchaeota* (Supplementary Table S1). In Nanoarchaeota, our analysis identified homologous sequences with similarity of between 26.82% and 40.32% and length alignment from 56% to 100% with the ancestral MβL fold and were detected in only *Nanoarchaeota archaeon* (Supplementary Table S2). Interestingly, while the MβL signature can be recognised within these sequences (Figure 2), almost all were annotated by default as MβL fold metallo-hydrolases (Supplementary Tables S1 and S2 and Figure 1).

### 4.3. Reported Activities of Archaeal MβL Fold Enzymes

Despite the wide presence and distribution of the MβL fold enzymes in the Archaea domain so far, few of these enzymes have been reported in the literature to have proven enzymatic activity such as the that described from the hyperthermophilic archaeon *Sulfolobus tokodaii* species [38]. Indeed, this *S. tokodaii* MβL fold enzyme, exhibiting protein structure similarity with the PqsE enzyme from *P. aeruginosa* involved in the quorum sensing mechanism [61], has been suggested as being involved in as yet undescribed quorum sensing mechanism in archaea using the quinolone antibiotic as a signalling factor [38] (Table 1). Another archaeal MβL fold enzyme from *Haloferax volcanii*, exhibiting sequence similarity with a MβL tRNase Z (a tRNA 3′-endonuclease) has been characterised from the transcriptome analysis of generated mutants [37] (Table 1). Despite its similarity with the tRNase Z enzyme, the *H. volcanii* MβL fold enzyme has not shown tRNA 3′ processing or exonuclease activity, although its activity associated with the membrane transport mechanism has been proven [37] (Table 1). In another archaeal species, *Methanosarcina mazei*, the reported enzyme was a MβL tRNase Z involved in tRNA maturation (cleavage and polyadenylation of the mRNA) [39] (Table 1). From the archaeal species, i.e., *Methanocaldococcus jannaschii*, three MβL fold enzymes have been reported with distinct enzymatic activities [41]. In the genome of this methanogenic archaeon *M. jannaschii*, the three genes (mjRNase J1, mjRNase J2, and mjRNase J3), as a result of the exhibited HxHxDH motif, have been recognised as members of the MβL fold superfamily and their characterisation revealed homologous proteins related to ribonuclease Rnase J enzymes, initially discovered in *Bacillus subtilis*, which are able to exhibit both endo- and 5′→3′ exo-ribonucleolytic activities [62]. Interestingly, their enzymatic characterisation demonstrates optimal activity at 60 °C and 5′→3′ exonucleolytic activity for purified mjRNase J1 and mjRNase J3 enzymes while mjRNase J2 protein exhibited endonuclease activity (degrade ssDNA substrate) [41]. Apart from these archaeal MβL fold enzymes with reported activity in the literature, the MβL sequences identified by our BlastP analysis using a common ancestor sequence, are annotated either as MβL fold metallo-hydrolases, hydroxyacylglutathione hydrolases (detoxification glyoxalase II enzymes), or L-ascorbate metabolism protein UlaG (Figure 1).

Interestingly, while all these archaeal enzymes are annotated as “metallo-β-lactamases”, none so far have been tested for their hydrolytic activity on β-lactam antibiotics. This was the goal of our recent study, in which the MβL sequence identified from *M. barkeri* (MetbaB) (Table 1) was synthesised, cloned into the *E. coli* BL21(DE3) strain, and expressed in order to evaluate its different putative enzymatic activities including β-lactamase, ribonuclease, nuclease, and glyoxalase [6]. Comparison of the three-dimensional (3D) structure of the expressed protein revealed its high structural similarity with the bacterial New Delhi metallo-β-lactamase (NDM-1) from *Klebsiella pneumonaie*. As expected, the expressed protein was able to significantly hydrolyse β-lactam substrates including nitrocefin (a chromogenic cephalosporin) and penicillin G. In addition to the β-lactamase activity which was detected, this *M. barkeri* MβL fold enzyme was also able to significantly hydrolyse bacterial and synthetic RNA substrates, while no nuclease activity was found [6]. As reported in this study, the conducted phylogenetic analysis reveals that the studied archaeal MβL fold protein appeared phylogenetically related to glyoxalase II enzymes. As a result of this relationship, the MetbaB enzyme was tested and weak glyoxalase activity was detected [6].

## 5. MβL Fold Enzymes in Eukaryotes

### 5.1. MβL Fold Enzymes Described in the Eukaryote Domain of Life

In the literature, MβL fold proteins such as hydroxyacylglutathione hydrolase (glyoxalase II), arylsulfatase, DNA and RNA interacting enzymes, nucleotide phosphodiesterases, and CMP-N-acetylneuraminate monooxygenases, have been identified in different eukaryotes including *Arabidopsis thaliana*, *Saccharomyces cerevisiae*, *Drosophila melanogaster*, *Bos taurus*, *Mus musculus*, and *Homo sapiens* [26,63]. In 2016, eighteen MβL fold enzymes had been mentioned in the literature as being present in human cells [44]. These enzymes are polycistronic proteins in which the MβL fold domain can be recognised as a result of their MβL signature, as shown in Figure 2. These 18 human MβLs (hMβLs) are reported in three groups of enzymes: Group 1 relates to the glyoxalase II subfamily and consists of seven enzymes (HAGH, HAGHL, ETHE1, LACTB2, MβLAC1, MβLAC2, and PNKD enzymes) and is constitutively involved in cellular detoxification processes; Group 2 relates to the DNA/RNA interacting subfamily of enzymes composed of nine enzymes (SNM1A, SNM1B, SNM1C, ELAC1, ELAC2, CPSF73, CPSF100, CPSF73L, and INTS9), and Group 3 relates to other hMβLs, in which two enzymes have been reported, namely the NAPE-PLD gene encoding for N-acyl-phosphatidylethanolamine-hydrolysing phospholipase D (NAPE-PLD), and the CMAH gene, encoding for cytidine monophospho-N-acetylneuraminic acid hydroxylase [44].

### 5.2. Reported Enzymatic Activities of Human MβL Fold Enzymes

Interestingly, in addition to their natural activities in human cells, some of these hMβLs, including the SNM1A and SMN1B enzymes, have been described alongside other enzymatic activities such as the hydrolase activity on anti-cancer drugs like mitomycin C and cisplatin [64,65]. Surprisingly, while the term “metallo-β-lactamase” has been used to annotate these proteins, even for human proteins, their activity against β-lactam antibiotics has thus far not been reported.

In the same way, as we did for archaea, we reported the evaluation of enzymatic activities of four selected hMβL proteins, as a result of their conserved MβL “HxHxDH” motif and histidine residues (H196 and H263), as seen in bacterial MβLs (Table 1) [43]. These four hMβL enzymes were the MΒLAC2 protein associated with the biosynthesis of B-cell exosomes [42], the endoribonuclease LACTB2 protein [66], SNM1A, and SNM1B, with a function of DNA cross-link repair enzymes (nucleases) [67]. In this study, the proteins which were synthetised and optimised for expression in *E. coli* BL21(DE3) strains were tested against β-lactam antibiotics and, as expected, while, no activity was detected for the LACTB2 protein, the MΒLAC2, SNM1A, and SNM1B proteins were able to significantly hydrolyse nitrocefin and penicillin G, and this activity was inhibited by a β-lactamase inhibitor (sulbactam) [43].

## 6. MβL Fold Enzymes in Giant Viruses

### 6.1. Discovery of MβL Fold Enzymes in Giant Viruses

With the existence of evidence of MβL fold enzymes in all domains of life, including Bacteria, Archaea, and Eukaryotes, as mentioned above, we similarly conducted, as part of our research into these MβL fold enzymes, a search for MβL fold enzymes in Giant viruses. By searching for the conserved MβL motif against a protein database of giant viruses retrieved from the NCBI (n = 72,993 proteins), we were able to identify fifteen confirmed MβL fold sequences (based on a protein length of at least 200 aa). Default protein annotations of these gvMβL sequences from the NCBI database included ribonuclease Z, the ribonuclease BN/tRNA processing enzyme, the MβL fold metallo-hydrolase/oxidoreductase superfamily, the metallo-β-lactamase superfamily protein, the Ankyrin repeat domain-containing protein, and proteins with unknown function. However, as shown in Figure 1, while most of these gvMβL sequences grouped together within the phylogenetic tree, interestingly three gvMβLs branched within bacterial MβLs, suggestive of a horizontal exchange between bacteria and giant viruses, especially *Pandoravirus dulcis* and *Pandoravirus salinus*. Two other gvMβLs appear to be clustered with sequences from humans and one gvMβL grouped with Asgard MβL fold sequences (Figure 1).

### 6.2. Description of Dual Enzymatic Activity of a Giant Virus MβL Fold Enzyme

To the best of our knowledge, no studies have yet reported the activities of these MβL fold enzymes from a giant virus. Thus, in our recent work, we characterised the enzymatic activities of a MβL fold protein from giant *Tupanvirus deep ocean* (TupBlac protein), naturally involved in translation mechanisms in giant viruses. Protein analysis using a conserved domain search (CD Search) tool [68] revealed its membership of the ribonuclease Z group (the tRNA-processing endonuclease enzymes) and conserved motif analysis revealed its MβL signature (HxHxDH motif) and conserved residues (H60-H62-H65) [45]. As described in that study, the expressed TupBlac protein from the *E. coli* BL21(DE3) strain and its enzymatic characterisation demonstrated a dual β-lactamase and ribonuclease activity, since the TupBlac protein was able to hydrolyse nitrocefin, penicillin G, and RNA substrate (of bacteria or *Acanthamoeba castellanii*) [45] (Table 1).

## 7. MβL Fold Enzymes in Candidate Phyla Radiation (CPR)

### 7.1. Wide Diversity of MβL Fold Enzymes in the CPR Domain

As described in 2015, as a result of the power of deep sequencing methods and bioinformatic analyses, a new branch of microorganisms from the tree of life was discovered and named Candidate Phyla Radiation (CPR) [69,70]. This group of microorganisms, in addition to their symbiotic lifestyle with bacteria, is characterised by their small size (100 to 300 nm), reduced genomes (≈1-Mb), the lack of biosynthetic pathways, and their abundance in all environments and various human microbiomes [46,69]. As recently reported, we investigated the existence of the MβL fold enzymes in this new domain of life as part of the investigation into the resistomes of CPR microorganisms. Indeed, the in silico analyses, based on BlastP and functional domain prediction of 4062 CPR genomes to look for the presence of antibiotic resistance (AR)-like enzymes revealed highly equipped microorganisms with more than 85 different AR-like enzymes against 14 different classes of antimicrobials including aminoglycosides, glycopeptides, and β-lactams [71]. Among these AR-like enzymes, especially β-lactam resistance proteins, the search for an MβL signature (HxHxDH) revealed their existence in this domain of life, as shown in Figure 2 for representative sequences. The default annotations of these CPR proteins from the NCBI database are mainly “MβL fold metallo-hydrolase”, “β-lactamase domain protein”, or “RNA-metabolising metallo-β-lactamase”. Interestingly, as shown in Figure 1, the identified MβL fold sequences in CPR microorganisms constitute one branch from the phylogenetic tree in which archaeal and Asgard sequences can be identified, and one of them, from *Sulfolobus tokodaii*, was reported to have a function associated with the quorum sensing mechanism [38]. This may suggest a native function of these CPR MβL fold enzymes, associated with communication processes with bacteria, with which they share an obligate symbiotic lifestyle, as reported in the literature [70].

### 7.2. Reported Enzymatic Activity of CPR MβL Fold Enzymes on β-Lactam Antibiotics

Despite the default annotation of these enzymes, which may suggest a hydrolytic activity on antibiotics or RNA/DNA substrates, no studies have thus far reported enzymatic characterisation of these proteins. Thus, after we reported the presence and wide distribution of the MβL fold sequences in CPR microorganisms, we experimentally expressed, purified, and tested the enzymatic activity of five selected β-lactamase proteins, including class A and class B metallo-β-lactamases from CPR, to evaluate their ability to hydrolyse various substrates including β-lactams, RNA, and DNA [46] (Table 1). The three-dimensional (3D) structural analysis of these five CPR β-lactamases confirms (with 100% confidence) their structural similarity with bacterial class A β-lactamase, metal-dependent hydrolases of the β-lactamase superfamily II, human metallo-β-lactamase containing protein 1, ribonuclease J1, and dual endo- and exonuclease enzymes [46]. This study is the first and only piece of research that has reported on the β-lactam and RNA hydrolysing activity of MβL fold proteins from CPR groups.

## 8. Discussion

**Figure 3:**
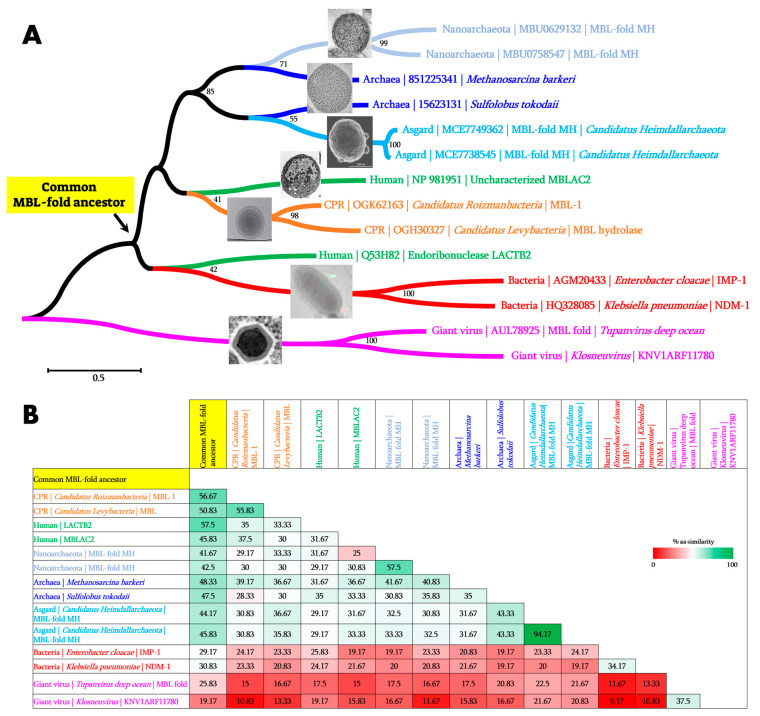
The phylogenetic tree and the pairwise comparison of protein similarity of representative MβL sequences from the different domains of life including Bacteria, Archaea, Asgard, Nanoarchaeota, Humans, Giant viruses, and Candidate phyla radiation (CPR). (**A**) Phylogenetic tree analysis using the Maximum likelihood (ML) method. From this tree, the common MβL fold ancestor has been inferred at the node indicated by arrow. (**B**) The pairwise comparison of the percentage of similarity of representative MβLs including the inferred MβL fold ancestor sequence. Pictures of each organism/microorganism have been retrieved from the literature [78,79,80,81,82,83].

**Figure 4:**
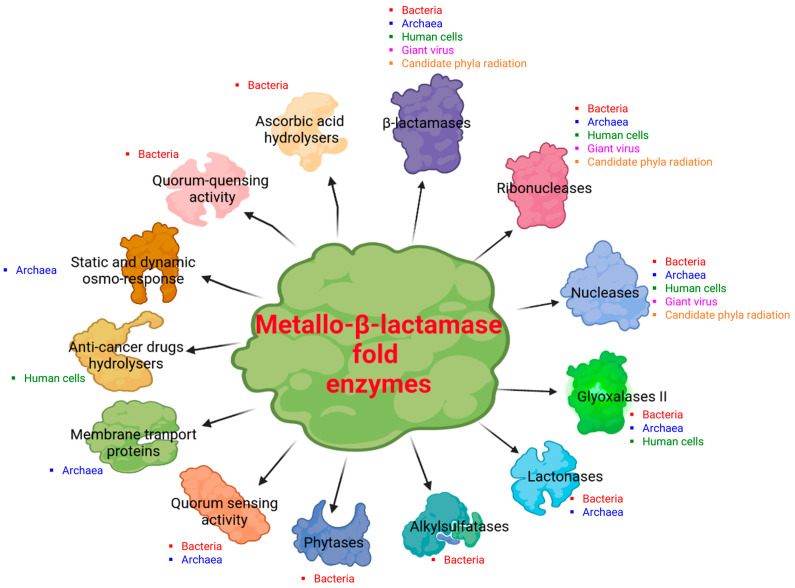
Schematic representation of the at least thirteen reported and demonstrated enzymatic or non-enzymatic activities of MβL fold proteins from the different domains of life. The figure was created in BioRender.com (https://app.biorender.com (accessed on 2 November 2022) and manually adapted.

In this review, we have highlighted the considerable extension of core genes for β-lactamases into all domains of life, as shown in Figure 3A. Metallo-β-lactamase fold proteins appear to be “multifunctional enzymes” with hydrolytic, non-hydrolytic, or non-enzymatic activities, as the literature reports at least thirteen demonstrated and distinct activities (Figure 4), and it is difficult to know what the initial activity was. It can be speculated that the primary activity was that of nuclease/ribonuclease, the first role of which was to digest unused or parasitic DNA/RNA in cellular metabolism as reported in bacteria, in humans, and in archaea [41,72,73]. The potential of these hydrolases was later used for different functions. β-lactamases are one of the most interesting examples in the history of science, as they were first discovered for their activity on β-lactam antibiotics, explaining bacterial resistance against these drugs [1,2]. It is plausible that the nomenclature of these enzymes has prevented the exploration of their real functions in organisms/microorganisms other than bacteria. β-lactamase fold sequences were recently identified for the first time in the human genome, while their default functions have been associated with various biological processes [44,66]. Indeed, the identification of genes encoding for β-lactamases in human genomes has not been explored for long, which has prevented the existence of β-lactamase activity in human cells that could inactivate penicillin G in these cells [43]. A great deal of confusion can be seen in the naming of these MβL fold enzymes as, according to the first Blast hit during the initial protein annotation, these latter are considered to have a unique and essential enzymatic activity instead taking into account other potential activities of these enzymes. For example, we initially had great difficulty in publishing the metallo-β-lactamase fold enzyme from archaea (from *Methanosarcina barkeri*) [6], given that it made no sense for archaea microorganisms to host β-lactamase enzymes, which were naturally resistant to β-lactams, based on the lack of β-lactam targets in their cell wall [74,75]. We can speculate that the existence or role of these MβL fold enzymes in archaea may be associated with other biological functions such as the RNA/DNA metabolism or the use of β-lactam antibiotics after enzymatic degradation as a source of carbon for nutrients, as described in some bacteria such as *Pseudomonas*, *Burkholderia,* and *Pandoreae* [76,77].

Our recent work highlights a wide distribution and great conservation of the MβL fold proteins in archaea [6]. By using a more sensitive search approach such as the use of common ancestor sequences in blast analysis, we can identify these MβL fold proteins in archaea-related microorganisms such as Nanoarchaeota and Asgard, as highlighted in this review (Figure 1). This approach demonstrates and confirms the power of using a common ancestor to identify distant homologous sequences hosted by unsuspected microorganisms as demonstrated for giant viruses for which the distance between the reconstructed RNA polymerase (RNAP) ancestor and RNAPs of bacteria, archaea, eukarya, and megavirales were significantly shorter than the distance between RNAPs from each organism [84]. Indeed, as can be seen in Figure 3B, the pairwise comparison performed on the similarity between MβL sequences from the different domains of life with the inferred common MβL fold ancestor sequence demonstrated a higher percentage of similarity between the ancestor MβL and sequences from the different organisms in comparison with any pairs of sequences from two different organisms.

## 9. Conclusions

The evidence of several activities including β-lactamase, nuclease, ribonuclease, lactonase, glyoxalase, phytase, and potentially other unidentified hydrolase activities from enzymes of the same family highlights the significance of accurately naming enzymes in order to understand their nuclear reactivity. This is particularly challenging due to the scarcity of competent reviewers outside the bacterial world able to evaluate knowledge extension beyond the initial enzyme activity identification, which remains extremely problematic. Classical Blast analyses present some limitations, for example, when it comes to identifying remote homologous sequences, because of the few similarities between them. This is why alternative search tools are now being proposed by NCBI to improve the shortcomings of the classical BlastP (i.e., PSI-BLAST: Position-Specific Iterated BLAST; PHI-BLAST: Pattern Hit Initiated BLAST; DELTA-BLAST: Domain Enhanced Lookup Time Accelerated BLAST). More sensitive search approaches including the search for Hidden Markov Models (HMM) profile, Sequence Similarity Network analysis (SSN), and the use of inferred common ancestor sequences as targets are more appropriate and more useful when it comes to detecting and identifying homologous sequences in any organism. This review highlights a great example of the misleading annotations of proteins in public sequence databases, which is not only limited to MβL fold proteins but applied to many classes of sequences proteins as previously reported, greatly reducing our perception of the multifunctionality of some proteins, due to the unique attributed function by the default protein annotation [85]. In essence, this history of β-lactamases has epistemological importance which we believe is essential.

## Figures and Tables

**Table 1:** β-lactamase fold enzymes with reported enzymatic activity from the different domains of life.

Kingdom	Acc. Number	Species	Gene Name	Size (aa)	Default Annotation	Reported Activities	References
**Bacteria**	AGM20433	*Enterobacter cloacae*	IMP-1	247	Imipenem hydrolysing β-lactamase	β-lactamase; Ribonuclease	[29]
	AEW99090	*Streptomyces cattleya*	ThnS	330	Putative β-lactamase	Imipenemase; Ascorbic acid degradation; Nuclease; Ribonuclease	[30]
	HQ328085	*Klebsiella pneumoniae*	NDM-1	270	New Delhi metallo-β-lactamase	β-lactamase; Ribonuclease	In this study
	NA	*Streptococcus pneumoniae*	-	363	L-ascorbate 6-phosphate lactonase	β-lactamase	[27]
	P39300.2	*Escherichia coli*	UlaG	354	L-ascorbate-6-phosphate lactonase	β-lactamase; Ribonuclease	[28]
	WP_010974862	*Agrobacterium tumefaciens*	AiiB	276	Zn-dependent hydrolases	Quorum-quenching lactonase	[33]
	PDB: 7EV5_A	*Bacillus lehensis*	BLEG-1	210	β-lactamase domain containing protein	β-lactamase; Glyoxalase II	[31]
	NA	Soil metagenome	MβLp01	312	Metallo-β-lactamase fold protein	β-lactamase; Phytase	[34]
	NA	Soil metagenome	MβLP02	355	Metallo-β-lactamase fold protein	β-lactamase; Phytase	[34]
	PDB: 7BZ4_B	Deep-seep sediments metagenome	PNGM-1	372	Metallo-β-lactamase	β-lactamase; Ribonuclease	[35]
	WP_063100708	*Escherichia coli*	YjcS	661	Uncharacterised protein	Alkyl sulfatase	[36]
**Archaea**	ELY35639	*Haloferax volcanii*	HVO_2763	278	Zn-dependent hydrolases of the β-lactamase fold	Membrane transport; Static and dynamic osmo-response	[37]
	851225341	*Methanosarcina barkeri*	MetbaB	214	MβL fold metallo-hydrolase	β-lactamase; Ribonuclease; and D-lactate hydrolase	[6]
	15623131	*Sulfolobus tokodaii*	-	200	Putative hydrolase	Quorum sensing activity	[38]
	AAM30391	*Methanosarcina mazei*	-	638	Metal-dependent RNase, contains metallo-β-lactamase and KH domains	Cleavage and Polyadenylation of mRNA	[39]
	BAD70075	*Thermus thermophilus*	TTHA0252		Metallo-β-lactamase superfamily	Ribonuclease (RNase)	[40]
	NA	*Methanocaldococcus jannaschii*	MjRNase J1		Ribonuclease Rnase J	Ribonuclease (RNase)	[41]
	NA	*Methanocaldococcus jannaschii*	MjRNase J2		Ribonuclease Rnase J	Ribonuclease (RNase); Nuclease	
	NA	*Methanocaldococcus jannaschii*	MjRNase J3		Ribonuclease Rnase J	Ribonuclease (RNase); Nuclease	
**Human**	NP_981951	*Homo sapiens*	MβLAC2	280	Metallo-β-lactamase domain	Exosome biogenesis enzyme; β-lactamase	[42,43]
	Q6PJP8	*Homo sapiens*	SNM1A	365	DNA cross-link repair 1A	Cisplatin or Mitomycin hydrolase; β-lactamase	[43,44]
	Q9H816	*Homo sapiens*	SNM1B	335	5′ exonuclease Apollo isoform X1	β-lactamase	[43]
**Giant viruses**	AUL78925	*Tupanvirus deep ocean*	TupBlac	322	β-lactamase superfamily domain	β-lactamase; Ribonuclease	[45]
**Candidate Phyla** **Radiation (CPR)**	KKR15801	*Candidatus Levybacteria*	-	287	β-lactamase class A-like protein	β-lactamase; Ribonuclease	[46]
	KKR17584	*Candidatus Levybacteria*	-	303	β-lactamase class A-like protein	β-lactamase; Ribonuclease	
	OGK62163	*Candidatus Roizmanbacteria*	-	263	Hypothetical protein	β-lactamase; Ribonuclease	
	OGZ64179	*Candidatus Staskawiczbacteria*	-	251	Hypothetical protein	β-lactamase; Ribonuclease	
	QHU90009	*Candidatus Saccharibacteria*	-	722	RNase J family beta-CASP ribonuclease	β-lactamase; Ribonuclease	

## Data Availability

Not applicable.

## Supplementary Materials

The following supporting information can be downloaded at: https://www.mdpi.com/article/10.3390/cells12131752/s1, Figure S1: Ribonuclease and nuclease activities of the New Delhi Metallo-β-lactamase 1 (NDM-1). (A) bacterial RNA substrate hydrolysed by the NDM-1 enzyme in the presence and absence of inhibitor (i.e., sulbactam and EDTA). (B) Single strand bacterial DNA (ssDNA forward and reverse) hydrolysed by the NDM-1 enzyme while double strand DNA (dsDNA) was not hydrolysed. H_2_O, Blank, and GO (Glycine oxidase) have been used as negative controls, and bacterial DNase has been used as positive control. The methodology and material used for this experiment have been reported in our previous work [30]. Table S1: BlastP result of the MβL fold ancestor (120 aa) sequence against the Asgard group database (NCBI) (94 hits); Table S2: BlastP results of the MβL fold ancestor (120 aa) against the Nanoarchaeota database (NCBI) (40 hits).
