# Supplementary material for: Origin, Diversity, and Multiple Roles of Enzymes with Metallo-β-Lactamase Fold from Different Organisms
Source: Cells. 2023 Jun 30;12(13):1752. doi: 10.3390/cells12131752 (PMC10340364; doi:10.3390/cells12131752)
Supplement: Supplementary file 1 [file cells-12-01752-s001.zip › Supplementary Figure S1.pdf]

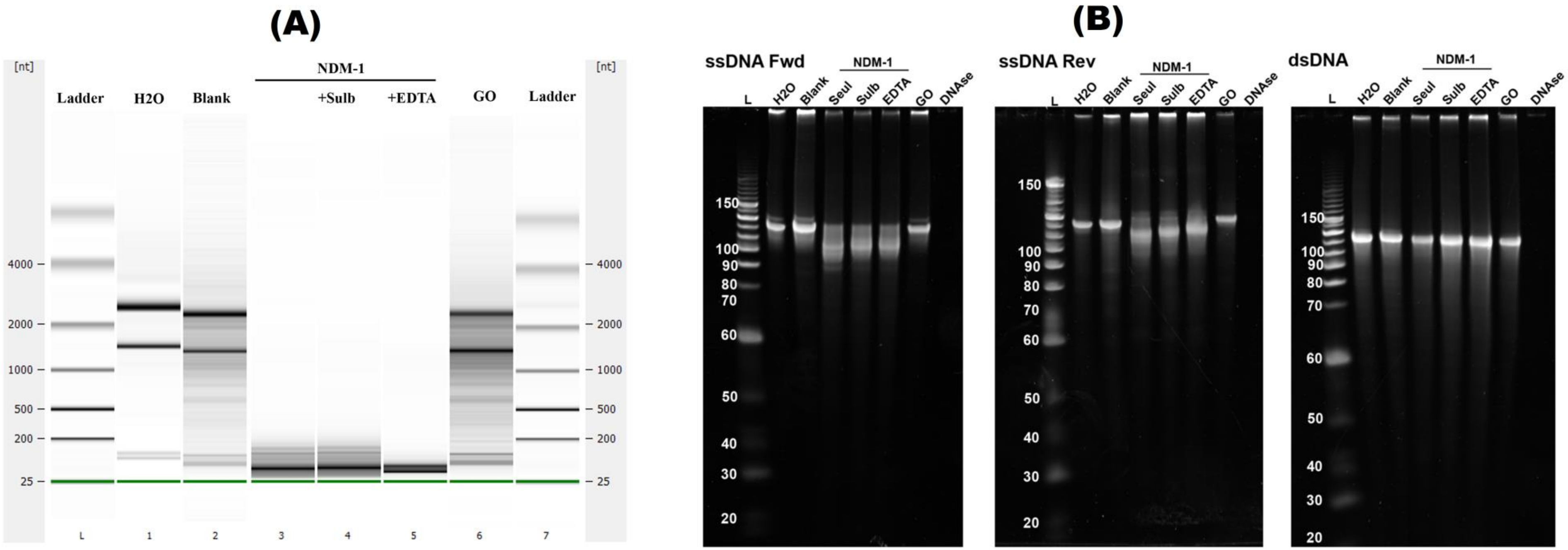

Supplementary Figure S1: Ribonuclease and nuclease activities of the New Delhi Metallo-β-lactamase 1 (NDM-1). (A): bacterial RNA substrate hydrolysed by the NDM-1 enzyme in the presence and absence of inhibitor (i.e., sulbactam and EDTA). (B): Single strand bacterial DNA (ssDNA forward and reverse) hydrolysed by the NDM-1 enzyme while double strand DNA (dsDNA) was not hydrolysed. H2O, Blank, and GO (Glycine oxidase) have been used as negative controls, and bacterial DNase has been used as positive control. The methodology and material used for this experiment have been reported in our previous work [30].
