# Supplementary material for: Origin, Diversity, and Multiple Roles of Enzymes with Metallo-β-Lactamase Fold from Different Organisms
Source: Cells. 2023 Jun 30;12(13):1752. doi: 10.3390/cells12131752 (PMC10340364; doi:10.3390/cells12131752)
Supplement: Supplementary file 1 [file cells-12-01752-s001.zip › Supplementary Table S1.pdf]

Supplementary Table S1: BlastP result of MBL Ancestor (120 aa) vs Asgard group database (NCBI) (94 hits)

Microorganism names	Predicted function	aa %Id	Query % cov.	E-value	Max Score	Hit size (aa)	Acc. Number	MBL signature
Candidatus Lokiarchaeota archaeon	MBL fold metallo-hydrolase	29,66	100%	6,00E-08	52	254	TFF89211	HsHvDH
Candidatus Helarchaeota archaeon	MBL fold metallo-hydrolase	28,98	100%	2,00E-11	60,8	206	NVM52811	HiHpDH
Candidatus Helarchaeota archaeon	MBL fold metallo-hydrolase	25	100%	3,00E-07	49,7	208	NVM29062	HvHpDH
Candidatus Heimdallarchaeota archaeon	MBL fold metallo-hydrolase	31,36	98%	2,00E-09	57	361	UCE14378	HtHaDH
Candidatus Lokiarchaeota archaeon	MBL fold metallo-hydrolase	29,67	96%	5,00E-09	55,1	268	MCK4369556	HeHfDH
Candidatus Lokiarchaeota archaeon	MBL fold metallo-hydrolase	36,3	95%	6,00E-11	59,7	200	NHI94306	HyHgDH
Candidatus Lokiarchaeota archaeon	MBL fold metallo-hydrolase	31,11	95%	5,00E-09	55,1	268	NVM35405	HeHfDH
Candidatus Lokiarchaeota archaeon	MBL fold metallo-hydrolase	30	95%	1,00E-08	53,9	268	TET58162	HeHfDH
Candidatus Lokiarchaeota archaeon	Zn-dependent hydrolase	30	95%	8,00E-09	54,7	268	TKJ21421	HeHfDH
Candidatus Lokiarchaeota archaeon	Zn-dependent hydrolase	27,72	95%	1,00E-06	48,1	219	TXT61116	HnHiDH
Candidatus Heimdallarchaeota archaeon	MBL fold metallo-hydrolase	33,15	94%	3,00E-14	68,6	210	MCG3216308	HlHfDH
Candidatus Heimdallarchaeota archaeon	MBL fold metallo-hydrolase	32,2	94%	2,00E-14	68,9	213	MCG3227015	HlHfDH
Asgard group archaeon	MBL fold metallo-hydrolase	31,07	94%	1,00E-12	64,3	212	NPD89532	HlHfDH
Candidatus Heimdallarchaeota archaeon	MBL fold metallo-hydrolase	30,9	94%	5,00E-13	65,5	213	MCE7738545	HlHfDH
Candidatus Heimdallarchaeota archaeon	MBL fold metallo-hydrolase	30,9	94%	1,00E-13	67	213	MCG3258853	HlHfDH
Candidatus Heimdallarchaeota archaeon	MBL fold metallo-hydrolase	28,89	94%	2,00E-09	55,8	207	UJG41838	HgHfDH
Candidatus Heimdallarchaeota archaeon	MBL fold metallo-hydrolase	28,65	94%	1,00E-09	56,6	214	MBY9000866	HlHfDH
Candidatus Heimdallarchaeota archaeon	MBL fold metallo-hydrolase	28,65	94%	5,00E-12	62,8	213	MCE7749362	HlHfDH
Candidatus Heimdallarchaeota archaeon	MBL fold metallo-hydrolase	28,25	94%	8,00E-12	62,4	212	MCG3222392	HlHfDH
Candidatus Heimdallarchaeota archaeon	MBL fold metallo-hydrolase	28,09	94%	3,00E-13	65,9	201	MCK4896778	HlHfDH
Candidatus Lokiarchaeota archaeon	MBL fold metallo-hydrolase	33,73	93%	1,00E-08	53,5	198	MBD3352654	HgHpDH
Candidatus Lokiarchaeota archaeon	MBL fold metallo-hydrolase	33,33	93%	5,00E-11	60,1	204	NHI94307	HiHpDH
Candidatus Lokiarchaeota archaeon	MBL fold metallo-hydrolase	32,93	93%	1,00E-09	56,2	198	MBY9013814	HgHsDH
Candidatus Lokiarchaeota archaeon CR_4	metallo-beta-lactamase protein	32,93	93%	4,00E-10	57,4	202	OLS12684	HgHpDH
Candidatus Helarchaeota archaeon	MBL fold metallo-hydrolase	32,72	93%	7,00E-13	65,1	208	NVM00699	HnHpDH
Candidatus Thorarchaeota archaeon	MBL fold metallo-hydrolase	31,49	93%	1,00E-11	62	211	NIU85394	HgHfDH
Candidatus Lokiarchaeota archaeon	MBL fold metallo-hydrolase	30,6	93%	4,00E-06	47	269	MCK4381340	HcHfDH

Candidatus Lokiarchaeota archaeon	MBL fold metallo-hydrolase	29,57	93%	9,00E-08	51,6	269	TFG28442	HcHwDH
Candidatus Lokiarchaeota archaeon	MBL fold metallo-hydrolase	28,57	93%	1,00E-06	48,1	220	MBY9011505	HlHiDH
Candidatus Thorarchaeota archaeon	MBL fold metallo-hydrolase	28,42	93%	8,00E-07	48,9	228	TFG04376	HsHiDH
Candidatus Lokiarchaeota archaeon	MBL fold metallo-hydrolase	28,02	93%	4,00E-06	47	220	MBY8980847	HfHiDH
Candidatus Lokiarchaeota archaeon	MBL fold metallo-hydrolase	27,98	93%	2,00E-08	53,1	216	TFG05538	HiHpDH
Candidatus Heimdallarchaeota archaeon	MBL fold metallo-hydrolase	27,93	93%	3,00E-08	52,8	207	UJG44655	HgHfDH
Candidatus Lokiarchaeota archaeon	MBL fold metallo-hydrolase	27,87	93%	2,00E-06	47,4	220	MBY9021610	HfHiDH
Candidatus Lokiarchaeota archaeon	MBL fold metallo-hydrolase	25,97	93%	9,00E-07	48,5	215	MBY8984334	HfHiDH
Candidatus Lokiarchaeota archaeon	MBL fold metallo-hydrolase	28,42	92%	3,00E-06	47,4	268	TFF63840	HfHwDH
Candidatus Heimdallarchaeota archaeon	MBL fold metallo-hydrolase	27,81	92%	2,00E-07	50,4	232	MCK5343769	HgHiDH
Candidatus Heimdallarchaeota archaeon	hypothetical protein	27,81	92%	1,00E-07	51,2	232	PWI49420	HgHiDH
Candidatus Heimdallarchaeota archaeon	MBL fold metallo-hydrolase	34,44	88%	8,00E-16	73,6	272	MCG3218260	HsHfDH
Candidatus Heimdallarchaeota archaeon	MBL fold metallo-hydrolase	30,77	88%	2,00E-06	48,1	282	MCK5047437	HsHyDH
Asgard group archaeon	MBL fold metallo-hydrolase	30,22	88%	4,00E-06	47	279	NPE08543	HsHyDH
Candidatus Heimdallarchaeota archaeon	MBL fold metallo-hydrolase	28,57	88%	2,00E-06	48,1	279	MCE7746212	HtHyDH
Candidatus Thorarchaeota archaeon	hypothetical protein	28,16	88%	5,00E-07	49,3	227	KXH71551	HsHiDH
Candidatus Thorarchaeota archaeon	MBL fold metallo-hydrolase	28,38	87%	7,00E-09	54,3	201	RLI51705	HhHsDH
Lokiarchaeum sp. GC14_75	Hydroxyacylglutathione hydrolase	28,79	86%	2,00E-06	48,1	469	KKK44737	HeHpDH
Candidatus Heimdallarchaeota archaeon	MBL fold metallo-hydrolase	31,39	85%	2,00E-08	53,9	361	UCG03848	HtHaDH
Candidatus Helarchaeota archaeon	MBL fold metallo-hydrolase	30,97	85%	3,00E-07	50,1	331	NVM27601	HsHiDH
Candidatus Lokiarchaeota archaeon	MBL fold metallo-hydrolase	29,76	85%	5,00E-10	57,4	220	TFG15931	HfHiDH
Candidatus Lokiarchaeota archaeon	MBL fold metallo-hydrolase	28,67	85%	7,00E-07	49,3	276	MBY9020760	HeHfDH
Candidatus Lokiarchaeota archaeon	MBL fold metallo-hydrolase	29,61	83%	8,00E-07	49,3	362	MBN1800984	HeHfDH
Candidatus Lokiarchaeota archaeon	MBL fold metallo-hydrolase	28,57	83%	8,00E-07	48,9	276	MBY9009056	HeHfDH
Candidatus Odinarchaeota archaeon	putative metallo-hydrolase	34,21	81%	4,00E-11	60,5	220	OLS17334	HvHfDH
Candidatus Lokiarchaeota archaeon	MBL fold metallo-hydrolase	31,39	81%	8,00E-09	53,9	210	MBD3226603	HkHpDH
Candidatus Thorarchaeota archaeon	MBL fold metallo-hydrolase	35,94	77%	4,00E-11	61,2	301	NIU82202	HtHgDH
Candidatus Lokiarchaeota archaeon	MBL fold metallo-hydrolase	33,08	75%	2,00E-09	55,8	210	TFF86283	HkHpDH
Candidatus Heimdallarchaeota	Beta-lactamase hydrolase-like	32,52	75%	4,00E-06	46,2	169	OLS32805	HsHaDH
Asgard group archaeon	MBL fold metallo-hydrolase	31,71	75%	2,00E-06	47,8	219	NPD89726	HsHaDH

Candidatus Heimdallarchaeota archaeon	MBL fold metallo-hydrolase	28,37	75%	8,00E-11	58,5	162	MCK5140869	HlHfDH
Candidatus Heimdallarchaeota archaeon	MBL fold metallo-hydrolase	28,37	75%	8,00E-11	58,9	163	MCK5304619	HlHfDH
Candidatus Heimdallarchaeota archaeon	MBL fold metallo-hydrolase	27,66	75%	1,00E-11	60,5	162	MCK4971958	HlHfDH
Candidatus Lokiarchaeota archaeon	MBL fold metallo-hydrolase	27,05	73%	3,00E-06	47,4	366	NMC07444	HtHaDH
Candidatus Lokiarchaeota archaeon	MBL fold metallo-hydrolase	42,17	59%	8,00E-08	52	349	MCK5588320	HsHaDH
Candidatus Helarchaeota archaeon	MBL fold metallo-hydrolase	42,67	58%	2,00E-13	67,4	297	NVM29969	HfHlDH
Candidatus Lokiarchaeota archaeon	MBL fold metallo-hydrolase	39,76	58%	5,00E-09	55,1	308	NHI94917	HwHvDH
Candidatus Thorarchaeota archaeon	MBL fold metallo-hydrolase	34,15	58%	4,00E-06	47	303	UCH04213	HiHpDH
Candidatus Lokiarchaeota archaeon	MBL fold metallo-hydrolase	42,11	57%	8,00E-10	57,4	323	TFF90670	HsHhDH
Lokiarchaeum sp. GC14_75	N-acyl homoserine lactonase	41,89	57%	4,00E-11	60,8	293	KKK46137	HfHaDH
Candidatus Helarchaeota archaeon	MBL fold metallo-hydrolase	46,25	56%	1,00E-10	58,9	207	NVM04758	HgHiDH
Lokiarchaeum sp. GC14_75	N-acyl homoserine lactonase	45,21	56%	1,00E-12	65,5	293	KKK40112	HfHvDH
Asgard group archaeon	MBL fold metallo-hydrolase	43,21	56%	4,00E-08	52,4	220	NHJ49411	HcHvDH
Candidatus Lokiarchaeota archaeon	MBL fold metallo-hydrolase	40,54	56%	1,00E-09	57	294	MBY8985829	HfHaDH
Candidatus Helarchaeota archaeon	MBL fold metallo-hydrolase	45,95	55%	4,00E-12	63,9	325	NVM03697	HsHvDH
Candidatus Lokiarchaeota archaeon	MBL fold metallo-hydrolase	44,3	55%	2,00E-09	55,5	206	MBD3340311	HgHvDH
Candidatus Lokiarchaeota archaeon	MBL fold metallo-hydrolase	43,24	55%	5,00E-07	49,7	323	MBD3227789	HsHhDH
Candidatus Helarchaeota archaeon	MBL fold metallo-hydrolase	43,21	55%	1,00E-08	53,9	244	NVM01246	HyHiDH
Candidatus Heimdallarchaeota archaeon	MBL fold metallo-hydrolase	42,5	55%	2,00E-07	50,8	250	TET76246	HgHvDH
Candidatus Lokiarchaeota archaeon	MBL fold metallo-hydrolase	41,89	55%	9,00E-07	48,9	328	NHI93006	HsHlDH
Candidatus Lokiarchaeota archaeon	MBL fold metallo-hydrolase	41,25	55%	3,00E-06	47,4	252	MBD3230696	HcHvDH
Candidatus Lokiarchaeota archaeon	MBL fold metallo-hydrolase	38,46	55%	2,00E-06	47,8	258	MBD3230458	HfHlDH
Candidatus Lokiarchaeota archaeon	MBL fold metallo-hydrolase	38,46	55%	2,00E-06	47,8	274	MBY9003930	HeHfDH
Candidatus Helarchaeota archaeon	MBL fold metallo-hydrolase	37,8	55%	1,00E-08	53,9	288	NVM56090	HlHrDH
Candidatus Lokiarchaeota archaeon	MBL fold metallo-hydrolase	37,18	55%	8,00E-07	48,9	277	NHJ21661	HeHfDH
Candidatus Lokiarchaeota archaeon	Hydroxyacylglutathione hydrolase	35,79	55%	8,00E-07	49,3	347	TXT65392	HnHlDH
Candidatus Lokiarchaeota archaeon	MBL fold metallo-hydrolase	35,56	55%	2,00E-06	48,1	340	TFG18023	HiHiDH
Candidatus Lokiarchaeota archaeon	MBL fold metallo-hydrolase	35,56	55%	7,00E-07	49,3	341	TFG25937	HiHtDH
Candidatus Lokiarchaeota archaeon	MBL fold metallo-hydrolase	35,56	55%	1,00E-06	48,9	340	UCC20058	HiHtDH
Candidatus Lokiarchaeota archaeon	MBL fold metallo-hydrolase	34,44	55%	1,00E-06	48,5	340	NVM38173	HlHtDH

Candidatus Lokiarchaeota archaeon	MBL fold metallo-hydrolase	33,33	55%	2,00E-06	47,8	340	NVM17664	HnHiDH
Candidatus Lokiarchaeota archaeon	MBL fold metallo-hydrolase	36,11	53%	2,00E-06	48,1	276	TFG20974	HeHfDH
Candidatus Lokiarchaeota archaeon	MBL fold metallo-hydrolase	37,93	52%	1,00E-07	51,2	343	TFG01099	HnHmDH
Candidatus Lokiarchaeota archaeon	MBL fold metallo-hydrolase	41,18	50%	5,00E-07	49,3	223	MBN1214050	HcHiDH
Candidatus Heimdallarchaeota archaeon	hypothetical protein	43,66	49%	1,00E-06	48,9	273	MBS85310	HnHfDH
Candidatus Lokiarchaeota archaeon	MBL fold metallo-hydrolase	42,42	49%	2,00E-07	50,8	332	TFG05878	HsHiDH
Candidatus Lokiarchaeota archaeon	MBL fold metallo-hydrolase	44,12	48%	5,00E-07	49,7	344	MBD3339864	HgHpDH
