# Supplementary material for: Origin, Diversity, and Multiple Roles of Enzymes with Metallo-β-Lactamase Fold from Different Organisms
Source: Cells. 2023 Jun 30;12(13):1752. doi: 10.3390/cells12131752 (PMC10340364; doi:10.3390/cells12131752)
Supplement: Supplementary file 1 [file cells-12-01752-s001.zip › Supplementary Table S2.pdf]

Supplementary Table S2: BlastP results of the MBL Ancestor (120 aa) against the Nanoarchaeota database (NCBI) (40 hits)

Scientific Name	Predicted functions	aa % Id	Query %cov.	E-value	Max Score	Hit Size (aa)	MBL signature
Nanoarchaeota archaeon	MBL fold metallo-hydrolase	30,59	56%	0,032	33,5	116	HiHpDH
Nanoarchaeota archaeon	MBL fold metallo-hydrolase	32,97	62%	0,01	35,8	256	HcHyDH
Nanoarchaeota archaeon	MBL fold metallo-hydrolase	30,15	75%	6,00E-09	52,8	194	HhHfDH
Nanoarchaeota archaeon	MBL fold metallo-hydrolase	35,82	75%	1,00E-06	46,6	193	HlHyDH
Nanoarchaeota archaeon	MBL fold metallo-hydrolase	36,45	75%	2,00E-09	53,5	165	HlHmDH
Nanoarchaeota archaeon	TPA: MBL fold metallo-hydrolase	37,74	75%	4,00E-13	61,6	108	HsHyDH
Nanoarchaeota archaeon	MBL fold metallo-hydrolase	37,72	77%	9,00E-14	64,3	141	HlHfDH
Candidatus Tiddalikarchaeum	Metallo-beta-lactamase fold protein	33,33	85%	5,00E-09	52,8	183	HlHpDH
Nanoarchaeota archaeon	MBL fold metallo-hydrolase	37,16	87%	3,00E-13	63,5	177	HaHfDH
Nanoarchaeota archaeon	MBL fold metallo-hydrolase	33,1	88%	5,00E-14	66,6	206	HnHyDH
Nanoarchaeota archaeon	MBL fold metallo-hydrolase	40,32	88%	4,00E-17	73,6	162	HnHyDH
Nanoarchaeota archaeon	MBL fold metallo-hydrolase	31,11	89%	6,00E-12	60,5	188	HnHfDH
Nanoarchaeota archaeon	MBL fold metallo-hydrolase	32,89	89%	4,00E-10	55,8	203	HlHfDH
Nanoarchaeota archaeon	MBL fold metallo-hydrolase	33,78	89%	3,00E-13	64,3	193	HlHyDH
Nanoarchaeota archaeon	MBL fold metallo-hydrolase	34,01	89%	5,00E-14	65,9	182	HsHwDH
Nanoarchaeota archaeon	MBL fold metallo-hydrolase	34,01	89%	7,00E-14	65,9	192	HlHfDH
Nanoarchaeota archaeon	MBL fold metallo-hydrolase	28,76	90%	2,00E-11	58,9	165	HnHfDH
Nanoarchaeota archaeon	MBL fold metallo-hydrolase	33,57	90%	1,00E-13	64,7	185	HlHyDH
Nanoarchaeota archaeon	MBL fold metallo-hydrolase	37,23	91%	5,00E-15	68,2	162	HfHfDH
Nanoarchaeota archaeon	MBL fold metallo-hydrolase	27,65	92%	6,00E-08	50,1	192	HlHyDH
Nanoarchaeota archaeon	MBL fold metallo-hydrolase	32	92%	2,00E-12	62	212	HeHtDH
Nanoarchaeota archaeon	MBL fold metallo-hydrolase	28,16	93%	7,00E-09	52,4	194	HlHyDH
Nanoarchaeota archaeon	MBL fold metallo-hydrolase	29,07	93%	1,00E-07	49,3	192	HfHfDH
Nanoarchaeota archaeon	MBL fold metallo-hydrolase	30,73	93%	4,00E-10	56,2	217	HtHfDH
Nanoarchaeota archaeon	MBL fold metallo-hydrolase	33,74	93%	4,00E-13	63,5	181	HcHfDH
Nanoarchaeota archaeon	MBL fold metallo-hydrolase	34,66	93%	4,00E-14	67	227	HaHfDH
Nanoarchaeota archaeon	MBL fold metallo-hydrolase	35,22	93%	5,00E-11	58,2	178	HeHfDH

Nanoarchaeota archaeon	MBL fold metallo-hydrolase	30,81	94%	6,00E-11	58,2	193	HnHyDH
Nanoarchaeota archaeon	MBL fold metallo-hydrolase	26,82	99%	7,00E-07	47	198	HlHyDH
Nanoarchaeota archaeon	hypothetical protein DRN75_01390	29,01	99%	5,00E-12	60,5	165	HlHyDH
Nanoarchaeota archaeon	MBL fold metallo-hydrolase	29,94	99%	8,00E-11	57,8	189	HlHyDH
Nanoarchaeota archaeon	MBL fold metallo-hydrolase	30,34	99%	4,00E-13	63,5	191	HlHyDH
Nanoarchaeota archaeon	MBL fold metallo-hydrolase	31,43	99%	1,00E-11	60,1	187	HtHfDH
Nanoarchaeota archaeon	MBL fold metallo-hydrolase	31,43	99%	1,00E-11	60,1	193	HsHfDH
Nanoarchaeota archaeon	MBL fold metallo-hydrolase	31,43	99%	5,00E-09	52,8	184	HcHfDH
Nanoarchaeota archaeon	MBL fold metallo-hydrolase	30,05	100%	2,00E-11	59,3	185	HsHfDH
Nanoarchaeota archaeon	MBL fold metallo-hydrolase	30,05	100%	1,00E-11	59,7	187	HcHcDH
Nanoarchaeota archaeon	MBL fold metallo-hydrolase	32,07	100%	2,00E-13	65,1	209	HlHlDH
Nanoarchaeota archaeon	MBL fold metallo-hydrolase	34,43	100%	4,00E-14	67	216	HlHfDH
Nanoarchaeota archaeon	MBL fold metallo-hydrolase	34,76	100%	5,00E-14	65,5	166	HdHwDH
